# Balloon aortic valvuloplasty with simultaneous aortic root injection: a case report of an adjunctive strategy to computed tomography for predicting coronary obstruction in transcatheter aortic valve-in-transcatheter aortic valve procedures

**DOI:** 10.1093/ehjcr/ytae622

**Published:** 2024-11-26

**Authors:** Tetsuro Shimura, Masanori Yamamoto, Hitoshi Matsuo

**Affiliations:** Department of Cardiology, Gifu Heart Center, 4-14-4 Yabuta-minami, Gifu-city, Gifu 500-8384, Japan; Department of Cardiology, Gifu Heart Center, 4-14-4 Yabuta-minami, Gifu-city, Gifu 500-8384, Japan; Department of Cardiology, Toyohashi Heart Center, 21-1 Gobutori, Oyama-cho, Toyohashi, Aichi 441-8530, Japan; Department of Cardiology, Nagoya Heart Center, 1-1-14 Sunadabashi, Higashi-ku, Nagoya, Aichi 461-2245, Japan; Department of Cardiology, Gifu Heart Center, 4-14-4 Yabuta-minami, Gifu-city, Gifu 500-8384, Japan

**Keywords:** TAV-in-TAV, Redo TAVI, Coronary obstruction, Sinus sequestration, Balloon aortic valvuloplasty, Simultaneous aortic root injection, Case report

## Abstract

**Background:**

Computed tomography (CT) assessment is the standard for predicting coronary obstruction (CO) caused by sinus sequestration (SS) during transcatheter aortic valve (TAV) implantation in degenerated TAV (TAV-in-TAV) procedure, but it may not always be accurate. This report describes a prediction method for CO by using balloon aortic valvuloplasty (BAV) during TAV-in-TAV.

**Case summary:**

An 87-year-old woman with a history of balloon-expandable transcatheter heart valve (BE-THV) implantation 7 years prior was admitted with worsening dyspnoea. Echocardiography revealed severe THV deterioration, and CT confirmed calcium proliferation in the THV. Our heart team decided to perform a TAV-in-TAV procedure using a 23-mm BE-THV. Preoperative CT imaging indicated an intermediate risk of CO. To evaluate CO risk more precisely, the top of a 20-mm balloon was positioned near the top of a BE-THV stent and inflated, followed by simultaneous aortic root injection (SARI). During SARI, contrast flowed into both coronary arteries, predicting a low risk of CO. Based on these findings, TAV-in-TAV was performed without coronary protection. The procedure was completed successfully without CO. After the procedure, the patient’s symptoms improved, and echocardiography showed normal valve function. She was discharged without complications and remains under outpatient follow-up care.

**Discussion:**

The diagnostic method for predicting CO using BAV with SARI could serve as a valuable adjunctive diagnostic tool in patients with an intermediate or high risk of SS anatomy after TAV-in-TAV. In such cases, this method may provide additional insights concerning precise CO risk and the indication of leaflet modification technique during TAV-in-TAV.

**Learning points**

- A diagnostic method for predicting coronary obstruction (CO) during transcatheter aortic valve (TAV) implantation (TAVI) in degenerated TAV (TAV-in-TAV), using balloon aortic valvuloplasty at the top of the transcatheter heart valve and simultaneous aortic root injection, maybe a useful procedure in patients considered to have intermediate- or high-risk anatomy of sinus sequestration (SS) after TAV-in-TAV.
- As TAVI becomes more common in younger patients, a need arises for methods to accurately estimate the risk of CO with SS in advance for future TAV-in-TAV procedures in this demographic.

## Introduction

Transcatheter aortic valve (TAV) implantation (TAVI) in degenerated TAV (TAV-in-TAV) is an alternative treatment option for patients requiring reintervention due to structural valve deterioration (SVD) of the previously implanted transcatheter heart valve (THV).^1,2^ For optimizing the TAV-in-TAV procedure, preprocedural planning is important in size selection and determining the risk of complications, including coronary obstruction (CO). Although CO is a rare complication, it significantly impacts prognosis and remains a major concern during the TAV-in-TAV procedure, including CO caused by sinus sequestration (SS).^3,4^ The most widely accepted method for preprocedural planning and CO risk assessment is computed tomography (CT)–based evaluation.^5,6^ However, few detailed study results from actual clinical practice exist regarding CO risk assessment for TAV-in-TAV, and CT imaging can produce metal artefacts that may slightly distort the true anatomy.

We describe a successful case of intraoperative CO risk assessment using balloon aortic valvuloplasty (BAV), with the balloon inflated and its top positioned near the top of the THV stent frame, followed by simultaneous aortic root injection (SARI) during the TAV-in-TAV.

## Summary figure

**Figure:**
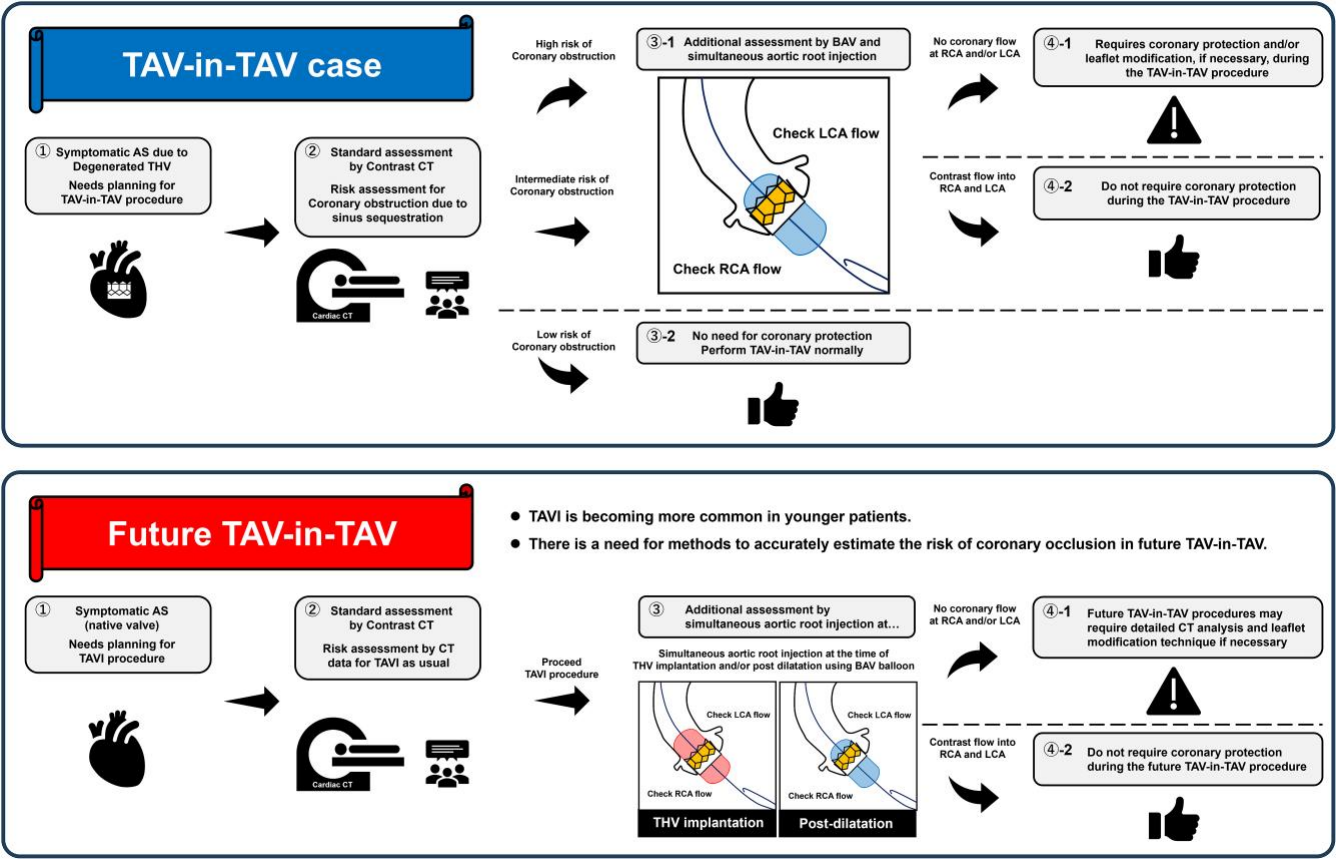


## Case presentation

An 87-year-old woman was admitted to our hospital with progressive dyspnoea and orthopnoea. She had undergone TAVI using a 23-mm SAPIEN3 (S3) valve (Edwards Lifesciences, Irvine, CA, USA) for severe aortic stenosis (AS) at our hospital 7 years prior. She also had a history of hypertension, chronic kidney disease, and diabetes mellitus, for which she was receiving outpatient medical therapy at another hospital. Her comorbidities were well controlled.

**Figure 1:**
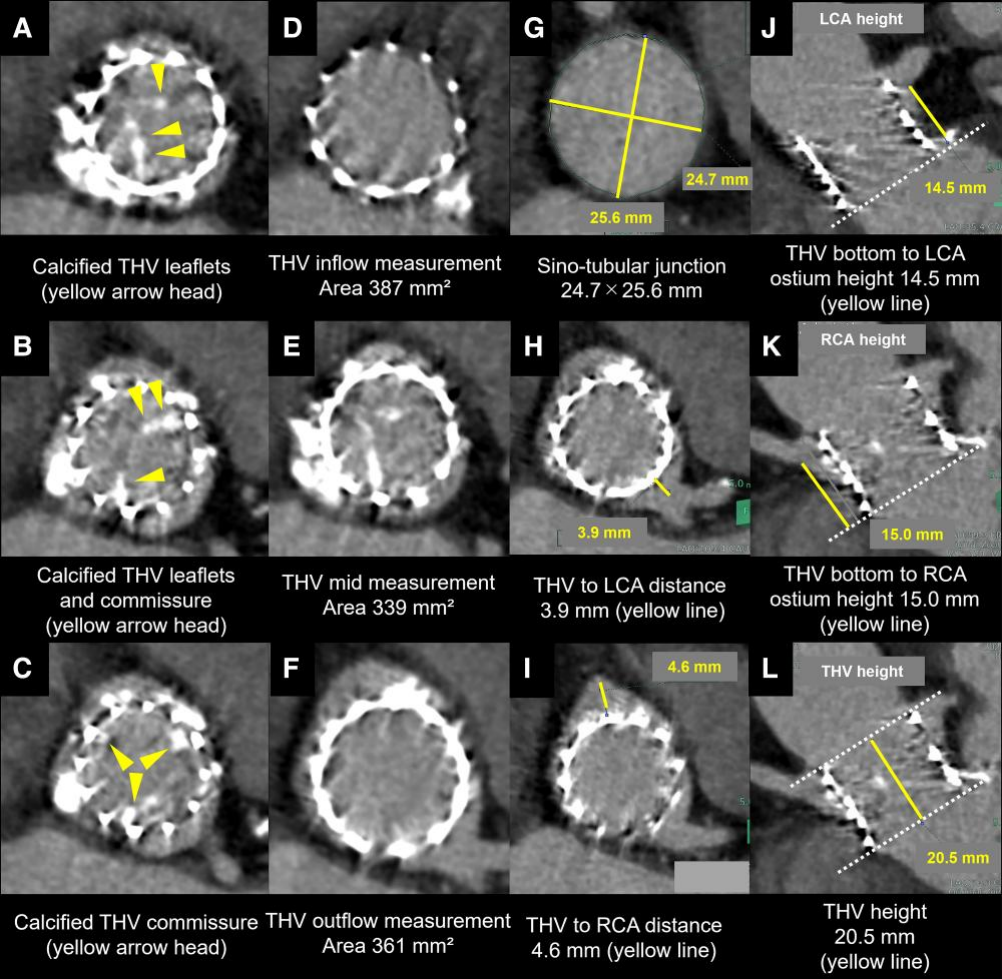
Contrast computed tomography revealed calcified transcatheter heart valve leaflets and commissures *(A–C)*. The transcatheter heart valve area at inflow was 387 mm^2^  *(D)*. The transcatheter heart valve area at mid-portion was 339 mm^2^  *(E)*. The transcatheter heart valve area at outflow was 361 mm^2^  *(F)*. The minimum and maximum sino-tubular junction diameters were 24.7 and 25.6 mm *(G)*, respectively. Distances to the left and right coronary arteries were 3.9 and 4.6 mm, respectively *(H and I)*. The distance of the lowest ostium of the left and right coronary arteries and the bottom of transcatheter heart valve were 14.5 and 15.0 mm, respectively *(J and K)*. The height of transcatheter heart valve was 20.5 mm *(L)*.

**Figure 2:**
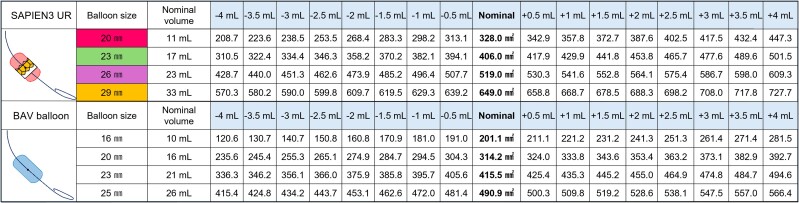
A detailed inflation size chart for the SAPIEN 3 ULTRA RESILIA and dedicated balloon aortic valvuloplasty balloon. The chart outlines the expansion area for each device in 0.5 mL increments, ranging from −4 to +4 mL.

**Figure 3:**
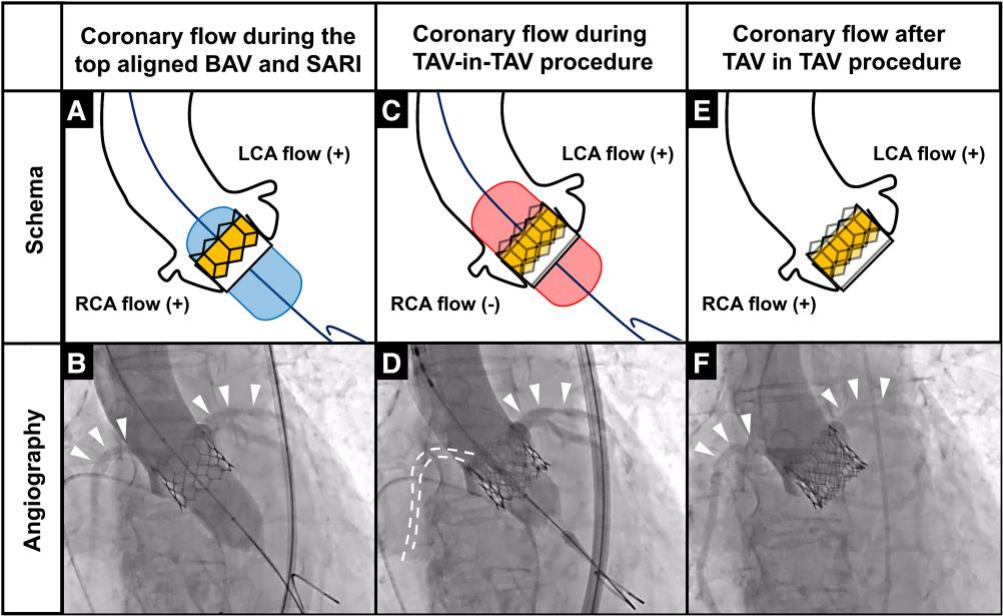
Schema of balloon aortic valvuloplasty with its top aligned at the top of the transcatheter heart valve *(A)*. Coronary flow was visualized during balloon aortic valvuloplasty with its top positioned near the top of the transcatheter heart valve with simultaneous aortic root injection *(B)*. Ballooning at the usual position during transcatheter aortic valve implantation can obstruct coronary flow *(C)*. The ballooning for transcatheter aortic valve implantation temporarily obstructed coronary flow *(D)*. Coronary flow is expected to resume after transcatheter aortic valve-in-transcatheter aortic valve *(E)*. Good coronary flow is observed *(F)*.

Upon admission, auscultation revealed a systolic cardiac murmur, and chest radiography revealed mild cardiomegaly without pulmonary congestion. Laboratory data showed a slightly elevated serum N-terminal pro-brain natriuretic peptide level (262 pg/mL). Transthoracic echocardiography showed a preserved left ventricular ejection fraction (52.8%), severe calcification of the THV leaflets, a peak flow velocity of 4.38 m/s, a mean pressure gradient of 35 mmHg, an aortic valve area of 0.34 cm², and mild to moderate aortic valve regurgitation (AR), indicating SVD of the previously implanted THV, manifesting as severe AS complicated by mild to moderate AR. The symptoms were attributed to SVD of the THV, while she did not develop acute heart failure, and her overall condition remained stable. A contrast-enhanced CT was subsequently performed, revealing calcified THV leaflets (*Figure 1A–C*), but no pannus formation was observed. The THV areas were 387, 339, and 361 mm² at the inflow, mid-portion, and outflow, respectively (*Figure 1D–F*). The sino-tubular junction diameters were 24.7 and 25.6 mm, respectively (*Figure 1G*). Distances to the left coronary artery (LCA) and right coronary artery (RCA) were 3.9 and 4.6 mm (*Figure 1H* and *I*), respectively. Distances of the lowest ostium of the LCA and RCA to the bottom of the THV stent were 14.5 and 15.0 mm, respectively, which were shorter than implanted THV stent height (*Figure 1J–L*), suggesting an intermediate risk of CO with SS during a TAV-in-TAV procedure.^6^ Given the patient’s advanced age and comorbidities, she was deemed to have a prohibitive surgical risk. Thus, our heart team opted for TAV-in-TAV using a 23-mm SAPIEN3 ULTRA RESILIA (S3UR) valve (Edwards Lifesciences, Irvine, CA, USA). *Figure 2* demonstrates the expected size of the S3UR and dedicated balloon for predilatation when adjusted for expansion capacity. The risk of CO was considered low if the contrast flowed into the LCA and RCA when the balloon was dilated with its top positioned near the top of the THV stent frame. In the present case, the minimum THV area was 339 mm^2^; it was expected that a dedicated 20-mm balloon for predilatation with a total of 17 mL (1 mL more than the normal volume capacity) that increased up to 333.8 mm^2^ would be close to the THV size. Balloon aortic valvuloplasty was performed using this 20-mm balloon with 1 mL more volume overfilling, followed by SARI (*Figure 3A*). Balloon aortic valvuloplasty performed with its top positioned near the top of the THV stent frame showed an inflow of contrast into the LCA and RCA (*Figure 3B*) (see Supplementary material online, *Video S1*), indicating a low CO risk; therefore, we proceeded without coronary protection. During S3UR implantation, blood flow to the RCA was temporarily lost (*Figure 3C* and *D*) (see Supplementary material online, *Video S2*) but resumed after balloon deflation, with final angiography showing no CO (*Figure 3E* and *F*) (see Supplementary material online, *Video S3*).

The patient’s symptoms improved significantly as a result of her favourable recovery. She was discharged 9 days after the TAV-in-TAV procedure. Four months post-treatment, the patient continues outpatient care without any worsening of symptoms or heart failure.

## Discussion

This is the first report describing a diagnostic support strategy for predicting CO due to SS during TAV-in-TAV using an inflated BAV balloon with its top positioned near the top of the THV stent frame, followed by SARI. The standard assessment method relies on CT data, which lacks validation with real practice and may be affected by metal artefacts, complicating precise evaluations.^5,6^ While approximate predictions can be made, detailed and accurate assessments remain difficult. In cases where a detailed assessment is required, combining CT with additional diagnostic methods may enhance accuracy in CO risk assessments during TAV-in-TAV.

Our approach to assessing CO risk in TAV-in-TAV involves pre-BAV with its top positioned near the top of the THV stent frame along with SARI (*Summary figure*). This BAV positioning allows for contrast injection in the aortic root without obstruction, even when the THV leaflet is compressed around the entire circumference of the THV stent frame during predilatation. This method simulates a situation similar to that of CO caused by SS after TAV-in-TAV. Unlike CT assessment, this approach may not be a minimally invasive or low-cost method that can be recommended for all TAV-in-TAV cases. Predilatation of a THV with SVD should be approached cautiously, particularly due to the risks of stroke and acute AR. Although valve thrombosis, pannus formation, or severe calcified degeneration was not present in our patient, these conditions may elevate the risk of stroke and/or AR. In such instances, a thorough risk-benefit assessment is crucial before proceeding with the procedure. Moreover, this method can mainly be applied when a balloon-expandable THV is used for TAV-in-TAV. However, the current method provides a significant advantage, as CT cannot assess blood flow to the coronary arteries during the TAV-in-TAV procedure. It could serve as a valuable adjunctive diagnostic tool in situations, such as the present case, where the risk of CO is considered intermediate or high based on CT assessment. In the present case, we proceeded with the TAV-in-TAV procedure without coronary protection, guided by the favourable outcomes of this approach. A more detailed sizing chart will allow the selection of the appropriate balloon size during SARI and a more accurate prediction of the CO risk after TAV-in-TAV. Recently, leaflet modification has been aggressively used to prevent CO associated with TAV-in-TAV.^7^ In patients with inadequate coronary artery visualisation during pre-BAV and SARI, it may be a good indicator to consider the leaflet modification before TAV-in-TAV.

This diagnostic method can also be applied during the TAV-in-TAV procedure and THV deployment or post-dilatation at the time of the first TAVI (*Summary figure*). As TAVI is increasingly performed in younger patients, post-dilatation with distal positioning during the initial TAVI offers the advantage of assessing the risk of future CO in advance for potential TAV-in-TAV procedures. Given that lifetime management of TAVI patients will become more important in the near future, additional methods for assessing the risk of future TAV-in-TAV during the initial TAVI procedure, such as the present approach, should be explored.

## Lead author biography


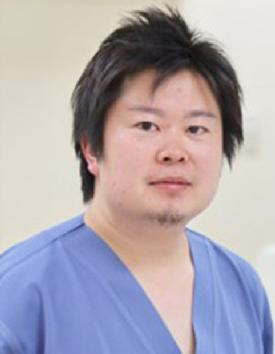


Tetsuro Shimura is an interventional cardiologist who graduated from Nippon Medical School, Tokyo, Japan, in 2009. Since October 2022, he has worked as chief of structural heart disease intervention at Gifu Heart Center (Gifu, Japan).

## Supplementary Material

ytae622_Supplementary_Data

## Data Availability

The data underlying this article cannot be shared publicly due to privacy and ethical reasons of individuals that participated in the study.
